# (1*R*,6*R*)-1-Methyl-8-aza­spiro­[5.6]dodecan-7-one

**DOI:** 10.1107/S1600536808015158

**Published:** 2008-05-24

**Authors:** Stéphanie M. Guéret, Ka Wai Choi, Patrick D. O’Connor, Peter D. W. Boyd, Margaret A. Brimble

**Affiliations:** a Department of Chemistry, Univerisity of Auckland, Private Bag 92019, Auckland, New Zealand

## Abstract

The crystal structure of the title compound, C_12_H_21_NO, has been investigated to establish the absolute stereochemistry at position 1. The absolute stereochemistry at the quaternary centre at position 6 is established to be *R* using an asymmetric Birch reductive alkyl­ation reaction for which the stereochemical outcome is known. The crystal structure indicates the presence of two conformers of the bicyclic (1*R*,6*R*)-spiro­lactam ring system that differ in the conformation adopted by the six-membered ring. In one conformer, the meth­yl group adopts an axial position whereas in the other conformer, the same methyl group adopts an equatorial position. In both conformers, the seven-membered ring adopts a chair conformation. The two conformers of the bicyclic spiro­lactam are connected to each other *via* inter­molecular N—H⋯O hydrogen bonds forming a heterodimer. The asymmetric unit contains two such dimers.

## Related literature

For related literature, see: Brimble & Trzoss (2004 ▶); Brimble *et al.* (2005 ▶); Ciminiello *et al.* (2007 ▶); Hu *et al.* (2001 ▶); MacKinnon *et al.* (2006 ▶); Schultz & Pettus (1997 ▶); Schultz *et al.* (1988 ▶).
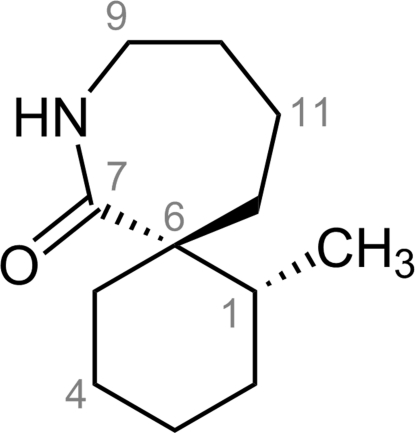


## Experimental

### 

#### Crystal data

C_12_H_21_NO
*M* *_r_* = 195.30
Triclinic, 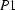

*a* = 8.5417 (2) Å
*b* = 10.2807 (2) Å
*c* = 12.6400 (3) Å
α = 102.850 (1)°
β = 90.091 (1)°
γ = 91.488 (1)°
*V* = 1081.78 (4) Å^3^
*Z* = 4
Mo *K*α radiation
μ = 0.07 mm^−1^
*T* = 90 (2) K
0.34 × 0.26 × 0.23 mm

#### Data collection

Bruker SMART diffractometer with APEX2 CCD detector
Absorption correction: none
25392 measured reflections
5160 independent reflections
4784 reflections with *I* > 2σ(*I*)
*R*_int_ = 0.035

#### Refinement

*R*[*F* ^2^ > 2σ(*F* ^2^)] = 0.040
*wR*(*F* ^2^) = 0.104
*S* = 1.02
5160 reflections
509 parameters
3 restraints
H-atom parameters constrained
Δρ_max_ = 0.46 e Å^−3^
Δρ_min_ = −0.21 e Å^−3^

### 

Data collection: *SMART* (Siemens, 1995 ▶); cell refinement: *SAINT* (Siemens, 1995 ▶); data reduction: *SAINT*; program(s) used to solve structure: *SHELXS97* (Sheldrick, 2008 ▶); program(s) used to refine structure: *SHELXL97* (Sheldrick, 2008 ▶); molecular graphics: *ORTEPIII* (Burnett & Johnson, 1996 ▶); software used to prepare material for publication: *SHELXTL* (Sheldrick, 2008 ▶) and *publCIF* (Westrip, 2008 ▶).

## Supplementary Material

Crystal structure: contains datablocks global, I. DOI: 10.1107/S1600536808015158/dn2350sup1.cif

Structure factors: contains datablocks I. DOI: 10.1107/S1600536808015158/dn2350Isup2.hkl

Additional supplementary materials:  crystallographic information; 3D view; checkCIF report

## Figures and Tables

**Table 1:** Hydrogen-bond geometry (Å, °)

*D*—H⋯*A*	*D*—H	H⋯*A*	*D*⋯*A*	*D*—H⋯*A*
N8—H8⋯O1*B*	0.86	2.11	2.967 (2)	173
N8*B*—H8*B*⋯O1	0.86	2.03	2.868 (2)	166
N8*A*—H8*A*⋯O1*C*	0.86	2.03	2.872 (2)	165
N8*C*—H8*C*⋯O1*A*	0.86	2.10	2.959 (2)	172

**Fig. 1.**
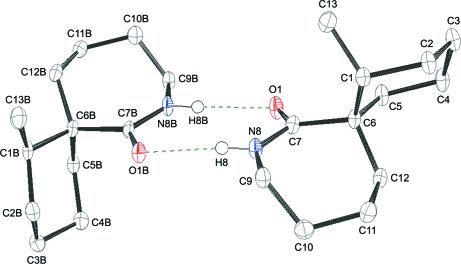
The molecular structure and atom numbering scheme of the heterodimer of (1R,6R)-1-methyl-8-azaspiro[5.6]dodecan-7-one. The respective methyl group (C13) adopts an axial or equatorial position on its associated cyclohexane ring as shown. Ellipsoids are drawn at the 50% probability level. H atoms are represented as small spheres of arbitrary radii. Hydrogen bonds are shown as dashed lines.

## supplementary crystallographic information

### Comment

The title spirolactam was prepared as part of a synthetic program directed towards the synthesis of spirolides A and C that were isolated from the culture of a toxic clone of the dinoflagellate *Alexandrium ostenfeldii* (Hu *et al.*, 2001, MacKinnon *et al.*, 2006, Ciminiello *et al.*, 2007). The work demonstrates methodology to access enantiopure 7,6-spirolactams. The quaternary spirocyclic centre is generally considered a challenging stereocentre to be constructed in a stereoselective fashion in organic synthesis (Brimble *et al.*, 2005; Brimble & Trzoss, 2004). By employing (*S*)-methoxypyrrolidine as a chiral auxiliary, a highly diastereoselective Birch reductive alkylation (Schultz *et al.*, 1988 and Schultz & Pettus, 1997) furnished the alkylated product with the desired stereochemistry at the quaternary carbon which was then converted to the title spirolactam in several steps. Since the stereochemistry at C6 is known to be *R*, the absolute configuration at C1 has therefore been assigned as *R*.

The crystal structure indicates the presence of two conformers of the enantiopure bicyclic (1*R*,6*R*)-spirolactam. While in both conformers, the 7,6-bicyclic ring system adopts a chair-chair conformation, the methyl group (C13) adopts a differing position between the conformer. In one conformer, the methyl group (C13) adopts an axial position whereas in the other conformer, the same methyl group (C13B) adopts an equatorial position on their associated cyclohexane ring. In solution at room temperature, the two conformers are rapidly interconverting to each other as shown by the total lack of dynamic effects in the ^1^H NMR spectrum at 400 MHz.

Each unit cell contains two heterodimers of the two chair-configured conformers of the bicyclic spirolactam. In each dimer, the axial and equatorial conformers are connected to each other by two adjacent intermolecular N—H···O hydrogen bonds (Figure 1).

### Experimental

To (2'*S*,1*R*,2*R*)-2-methyl-1-(4'-aminobutane)-1-[{(2'- ethoxymethyl)pyrrolidinyl}carbonyl]-2,5-cyclohexane (47.9 mg, 0.2 mmol) in water (1.6 ml) was added concentrated HCl (1.6 ml) and the mixture was heated under reflux overnight. After cooling to room temperature, the mixture was concentrated *in vacuo* and dried in a freeze-drier. The crude amino acid salt was dissolved in CH_2_Cl_2_/DMF (14.4 ml, 2:1) and DIPEA (0.15 ml, 0.9 mmol) was added. This resultant mixture was added dropwise to a solution of (benzotriazole-1-yloxy)tripyrrolidinophosphonium hexafluorophosphate (229.0 mg, 0.4 mmol) and DMAP (53.8 mg, 0.4 mmol) in CH_2_Cl_2_/DMF (43.2 ml, 2:1) over 8.5 h using a syringe pump. After stirring for further 13 h, the mixture was concentrated *in vacuo*. The residual oil was dissolved in CH_2_Cl_2_ (50 ml) and washed with aqueous HCl solution (0.5 *M*, 2×50 ml). The combined aqueous layers were extracted with CH_2_Cl_2_ (60 ml). The combined organic layers were washed with saturated NaHCO_3_ solution (60 ml), dried over anhydrous MgSO_4_, filtered and concentrated *in vacuo*. Purification by flash chromatography (20:80→70:30 EtOAc-hexanes) afforded the *title compound* (7.8 mg, 26%) as a white solid. Recrystallization from CH_2_Cl_2_ afforded white prisms.

M. P. 392.4–393.4 K.

HRMS (+EI) calculated for C_12_H_21_NO [*M*]^+^: 195.1623, found 195.1621.

IR (KBr plate neat) ν_max_ 3285, 2925, 2860, 1645, 1460, 1330, 1280, 1120 cm^-1^.

^1^H NMR (400 MHz, CDCl_3_) *δ* 5.96 (1*H*, s, CON*H*), 3.34 (1 H, m, NHC*H*_a_H_b_), 3.08 (1 H, m, NHCH_a_*H*_b_), 2.15 (3 H, m, 1-C*H* and 5-C*H*_a_H_b_ and 12-C*H*_a_H_b_), 1.74 (3*H*, m, 3-C*H*_a_H_b_, 5-CH_a_*H*_b_ and 10-C*H*_a_H_b_), 1.50 (6 H, m, 2-C*H*_a_H_b_ and 4-C*H*_a_H_b_ and 10-CH_a_*H*_b_ and 11-CH_2_ and 12-CH_a_*H*_b_), 1.32 (2 H, m, 3-CH_a_*H*_b_ and 4-CH_a_*H*_b_), 1.17 (1 H, m, 2-CH_a_*H*_b_), 1.00 (3*H*, d, J = 7.1 Hz, 13-C*H*_3_).

^13^C NMR (100 MHz, CDCl_3_) *δ* 181.0 (7-*C*O), 47.1 (6-*C*), 42.0 (9-N*C*H_2_), 31.5 (1-*C*H), 29.6 (5-*C*H_2_ and 2-*C*H_2_), 29.4 (12-*C*H_2_), 27.9 (3-*C*H_2_), 23.3 (10-*C*H_2_), 21.7 (11-*C*H_2_), 20.6 (4-*C*H_2_), 15.5 (13-*C*H_3_).

*m/z* (+EI, 70 eV) 195 ([*M*]^+^, 100), 180 (20), 166 (11), 140 (16%).

### Refinement

Hydrogen atoms were placed in calculated positions and refined using the riding model [C—H 0.93–0.97 Å), with *U*_iso_(H) = 1.5 *U*_eq_(C).

### Crystal data

**Table:**

C_12_H_21_NO	*Z* = 4
*M**_r_* = 195.30	*F*_000_ = 432
Triclinic, *P*1	*D*_x_ = 1.199 Mg m^−^^3^
Hall symbol: P 1	Melting point: 392.4 K
*a* = 8.5417 (2) Å	Mo *K*α radiation λ = 0.71073 Å
*b* = 10.2807 (2) Å	Cell parameters from 9917 reflections
*c* = 12.6400 (3) Å	θ = 1.7–28.0º
α = 102.850 (1)º	µ = 0.08 mm^−^^1^
β = 90.091 (1)º	*T* = 90 (2) K
γ = 91.488 (1)º	Prisms, white
*V* = 1081.78 (4) Å^3^	0.34 × 0.26 × 0.23 mm

### Data collection

**Table:**

Bruker SMART diffractometer with APEX2 CCD detector	4784 reflections with *I* > 2σ(*I*)
Radiation source: fine-focus sealed tube	*R*_int_ = 0.035
Monochromator: graphite	θ_max_ = 28.0º
*T* = 90(2) K	θ_min_ = 1.7º
ω scans	*h* = −11→10
Absorption correction: none	*k* = −13→13
25392 measured reflections	*l* = −16→16
5160 independent reflections	

### Refinement

**Table:**

Refinement on *F*^2^	Secondary atom site location: difference Fourier map
Least-squares matrix: full	Hydrogen site location: inferred from neighbouring sites
*R*[*F*^2^ > 2σ(*F*^2^)] = 0.040	H-atom parameters constrained
*wR*(*F*^2^) = 0.104	*w* = 1/[σ^2^(*F*_o_^2^) + (0.0667*P*)^2^ + 0.214*P*] where *P* = (*F*_o_^2^ + 2*F*_c_^2^)/3
*S* = 1.02	(Δ/σ)_max_ < 0.001
5160 reflections	Δρ_max_ = 0.46 e Å^−^^3^
509 parameters	Δρ_min_ = −0.21 e Å^−^^3^
3 restraints	Extinction correction: none
Primary atom site location: structure-invariant direct methods	

### Special details

**Table:**

Geometry. All esds (except the esd in the dihedral angle between two l.s. planes) are estimated using the full covariance matrix. The cell esds are taken into account individually in the estimation of esds in distances, angles and torsion angles; correlations between esds in cell parameters are only used when they are defined by crystal symmetry. An approximate (isotropic) treatment of cell esds is used for estimating esds involving l.s. planes.
Refinement. Refinement of *F*^2^ against ALL reflections. The weighted *R*-factor *wR* and goodness of fit *S* are based on *F*^2^, conventional *R*-factors *R* are based on *F*, with *F* set to zero for negative *F*^2^. The threshold expression of *F*^2^ > σ(*F*^2^) is used only for calculating *R*-factors(gt) *etc*. and is not relevant to the choice of reflections for refinement. *R*-factors based on *F*^2^ are statistically about twice as large as those based on *F*, and *R*- factors based on ALL data will be even larger. Friedel pairs were merged as recommended for light atom structures in the checkCIF program.

### Fractional atomic coordinates and isotropic or equivalent isotropic displacement parameters (Å^2^)

**Table:**

	*x*	*y*	*z*	*U*_iso_*/*U*_eq_	
C1	0.2813 (3)	0.3685 (2)	0.08818 (17)	0.0169 (4)	
H1	0.3372	0.3084	0.0303	0.020*	
C2	0.2355 (3)	0.2889 (2)	0.17364 (18)	0.0199 (4)	
H2A	0.1620	0.2176	0.1414	0.024*	
H2B	0.3282	0.2484	0.1953	0.024*	
C3	0.1618 (3)	0.3757 (2)	0.27424 (18)	0.0213 (5)	
H3A	0.0635	0.4092	0.2542	0.026*	
H3B	0.1398	0.3221	0.3270	0.026*	
C4	0.2710 (3)	0.4927 (2)	0.32488 (18)	0.0215 (5)	
H4A	0.3627	0.4594	0.3545	0.026*	
H4B	0.2178	0.5510	0.3841	0.026*	
C5	0.3225 (3)	0.5725 (2)	0.24137 (17)	0.0189 (4)	
H5A	0.3982	0.6411	0.2752	0.023*	
H5B	0.2323	0.6167	0.2205	0.023*	
C6	0.3959 (3)	0.4866 (2)	0.13778 (16)	0.0159 (4)	
C7	0.4210 (3)	0.5784 (2)	0.05733 (17)	0.0165 (4)	
C9	0.5649 (3)	0.4056 (2)	−0.08228 (18)	0.0189 (4)	
H9A	0.6043	0.4063	−0.1541	0.023*	
H9B	0.4854	0.3350	−0.0901	0.023*	
C10	0.6984 (3)	0.3737 (2)	−0.01351 (19)	0.0210 (4)	
H10A	0.7681	0.4514	0.0062	0.025*	
H10B	0.7576	0.3017	−0.0565	0.025*	
C11	0.6438 (3)	0.3333 (2)	0.08969 (19)	0.0214 (4)	
H11A	0.7346	0.3083	0.1261	0.026*	
H11B	0.5756	0.2547	0.0693	0.026*	
C12	0.5571 (3)	0.4396 (2)	0.17042 (18)	0.0184 (4)	
H12A	0.6262	0.5178	0.1894	0.022*	
H12B	0.5417	0.4065	0.2359	0.022*	
C13	0.1352 (3)	0.4142 (2)	0.0369 (2)	0.0234 (5)	
H13A	0.0696	0.3379	0.0061	0.035*	
H13B	0.1664	0.4590	−0.0190	0.035*	
H13C	0.0783	0.4742	0.0916	0.035*	
C1A	0.0229 (3)	0.4909 (2)	0.74816 (17)	0.0182 (4)	
H1A	0.0141	0.4245	0.7928	0.022*	
C2A	0.1973 (3)	0.5265 (2)	0.74572 (18)	0.0202 (4)	
H2A1	0.2118	0.5969	0.7065	0.024*	
H2A2	0.2346	0.5605	0.8195	0.024*	
C3A	0.2950 (3)	0.4080 (2)	0.69248 (19)	0.0225 (5)	
H3A1	0.4024	0.4379	0.6857	0.027*	
H3A2	0.2947	0.3431	0.7376	0.027*	
C4A	0.2290 (3)	0.3432 (2)	0.58066 (19)	0.0217 (4)	
H4A1	0.2886	0.2650	0.5496	0.026*	
H4A2	0.2397	0.4055	0.5334	0.026*	
C5A	0.0563 (3)	0.3023 (2)	0.58674 (18)	0.0185 (4)	
H5A1	0.0470	0.2366	0.6310	0.022*	
H5A2	0.0188	0.2606	0.5144	0.022*	
C6A	−0.0490 (3)	0.4215 (2)	0.63499 (17)	0.0161 (4)	
C7A	−0.0507 (3)	0.5153 (2)	0.55546 (17)	0.0156 (4)	
C9A	−0.2115 (3)	0.3552 (2)	0.40755 (18)	0.0190 (4)	
H9A1	−0.1394	0.2822	0.3965	0.023*	
H9A2	−0.2472	0.3639	0.3366	0.023*	
C10A	−0.3520 (3)	0.3193 (2)	0.47040 (18)	0.0212 (4)	
H10C	−0.4149	0.3976	0.4937	0.025*	
H10D	−0.4162	0.2516	0.4228	0.025*	
C11A	−0.3040 (3)	0.2678 (2)	0.56903 (19)	0.0208 (4)	
H11C	−0.2377	0.1918	0.5455	0.025*	
H11D	−0.3972	0.2368	0.6008	0.025*	
C12A	−0.2166 (3)	0.3715 (2)	0.65661 (18)	0.0197 (4)	
H12C	−0.2107	0.3350	0.7209	0.024*	
H12D	−0.2816	0.4493	0.6747	0.024*	
C13A	−0.0654 (3)	0.6130 (2)	0.81015 (18)	0.0226 (5)	
H13D	−0.0251	0.6394	0.8829	0.034*	
H13E	−0.1749	0.5903	0.8121	0.034*	
H13F	−0.0513	0.6853	0.7741	0.034*	
C1B	0.5341 (3)	0.8408 (2)	−0.36794 (16)	0.0178 (4)	
H1B	0.5290	0.9070	−0.4128	0.021*	
C2B	0.7072 (3)	0.8067 (2)	−0.36470 (18)	0.0199 (4)	
H2B1	0.7181	0.7366	−0.3251	0.024*	
H2B2	0.7428	0.7725	−0.4382	0.024*	
C3B	0.8108 (3)	0.9260 (2)	−0.31153 (19)	0.0209 (4)	
H3B1	0.8137	0.9908	−0.3568	0.025*	
H3B2	0.9168	0.8971	−0.3044	0.025*	
C4B	0.7482 (3)	0.9906 (2)	−0.19965 (18)	0.0203 (4)	
H4B1	0.8119	1.0693	−0.1685	0.024*	
H4B2	0.7554	0.9286	−0.1523	0.024*	
C5B	0.5778 (3)	1.0301 (2)	−0.20684 (17)	0.0180 (4)	
H5B1	0.5723	1.0955	−0.2514	0.022*	
H5B2	0.5423	1.0720	−0.1347	0.022*	
C6B	0.4660 (3)	0.9104 (2)	−0.25522 (17)	0.0153 (4)	
C7B	0.4590 (3)	0.8169 (2)	−0.17528 (16)	0.0152 (4)	
C9B	0.3049 (3)	0.9764 (2)	−0.02876 (18)	0.0185 (4)	
H9B1	0.3806	1.0502	−0.0171	0.022*	
H9B2	0.2677	0.9675	0.0418	0.022*	
C10B	0.1677 (3)	1.0105 (2)	−0.09283 (19)	0.0207 (4)	
H10E	0.1063	1.0777	−0.0460	0.025*	
H10F	0.1011	0.9314	−0.1166	0.025*	
C11B	0.2195 (3)	1.0621 (2)	−0.19119 (18)	0.0191 (4)	
H11E	0.2901	1.1385	−0.1673	0.023*	
H11F	0.1284	1.0925	−0.2237	0.023*	
C12B	0.3016 (3)	0.9586 (2)	−0.27761 (18)	0.0184 (4)	
H12E	0.3094	0.9945	−0.3422	0.022*	
H12F	0.2325	0.8803	−0.2955	0.022*	
C13B	0.4399 (3)	0.7182 (2)	−0.43024 (18)	0.0226 (5)	
H13G	0.4805	0.6909	−0.5023	0.034*	
H13H	0.3319	0.7404	−0.4340	0.034*	
H13I	0.4485	0.6466	−0.3933	0.034*	
C1C	−0.2016 (3)	0.9626 (2)	0.29179 (17)	0.0170 (4)	
H1C	−0.1435	1.0228	0.3506	0.020*	
C2C	−0.2420 (3)	1.0427 (2)	0.20650 (17)	0.0196 (4)	
H2C1	−0.3126	1.1129	0.2384	0.023*	
H2C2	−0.1470	1.0846	0.1864	0.023*	
C3C	−0.3183 (3)	0.9565 (2)	0.10453 (18)	0.0210 (4)	
H3C1	−0.4190	0.9219	0.1228	0.025*	
H3C2	−0.3361	1.0107	0.0521	0.025*	
C4C	−0.2146 (3)	0.8405 (2)	0.05437 (18)	0.0206 (4)	
H4C1	−0.1205	0.8749	0.0260	0.025*	
H4C2	−0.2700	0.7823	−0.0057	0.025*	
C5C	−0.1689 (3)	0.7601 (2)	0.13766 (17)	0.0192 (4)	
H5C1	−0.0964	0.6922	0.1042	0.023*	
H5C2	−0.2618	0.7150	0.1573	0.023*	
C6C	−0.0923 (3)	0.8458 (2)	0.24222 (16)	0.0152 (4)	
C7C	−0.0736 (3)	0.7534 (2)	0.32233 (17)	0.0172 (4)	
C9C	0.0785 (3)	0.9260 (2)	0.46415 (18)	0.0195 (4)	
H9C1	0.1166	0.9250	0.5363	0.023*	
H9C2	0.0033	0.9964	0.4713	0.023*	
C10C	0.2153 (3)	0.9581 (2)	0.39660 (19)	0.0223 (5)	
H10G	0.2795	0.8801	0.3766	0.027*	
H10H	0.2793	1.0293	0.4405	0.027*	
C11C	0.1644 (3)	1.0004 (2)	0.29360 (19)	0.0226 (5)	
H11G	0.1011	1.0788	0.3143	0.027*	
H11H	0.2572	1.0259	0.2581	0.027*	
C12C	0.0718 (3)	0.8950 (2)	0.21178 (18)	0.0188 (4)	
H12G	0.1366	0.8173	0.1921	0.023*	
H12H	0.0586	0.9294	0.1469	0.023*	
C13C	−0.3507 (3)	0.9146 (2)	0.3408 (2)	0.0236 (5)	
H13J	−0.4126	0.9900	0.3719	0.035*	
H13K	−0.3226	0.8688	0.3962	0.035*	
H13L	−0.4101	0.8549	0.2850	0.035*	
N8	0.4924 (2)	0.53304 (18)	−0.03820 (15)	0.0186 (4)	
H8	0.4959	0.5884	−0.0802	0.022*	
N8A	−0.1270 (2)	0.47750 (18)	0.45948 (15)	0.0187 (4)	
H8A	−0.1254	0.5375	0.4216	0.022*	
N8B	0.3840 (2)	0.85451 (18)	−0.07988 (15)	0.0180 (4)	
H8B	0.3826	0.7949	−0.0417	0.022*	
N8C	−0.0011 (2)	0.79845 (18)	0.41869 (15)	0.0184 (4)	
H8C	−0.0018	0.7428	0.4604	0.022*	
O1	0.3740 (2)	0.69436 (15)	0.07917 (13)	0.0225 (3)	
O1A	0.01941 (19)	0.62531 (15)	0.57620 (12)	0.0188 (3)	
O1B	0.52257 (19)	0.70723 (15)	−0.19575 (12)	0.0182 (3)	
O1C	−0.1266 (2)	0.63695 (15)	0.29949 (13)	0.0222 (3)	

### Atomic displacement parameters (Å^2^)

**Table:**

	*U*^11^	*U*^22^	*U*^33^	*U*^12^	*U*^13^	*U*^23^
C1	0.0211 (10)	0.0169 (10)	0.0134 (10)	0.0004 (8)	0.0009 (8)	0.0048 (8)
C2	0.0296 (12)	0.0158 (10)	0.0151 (10)	−0.0021 (8)	0.0021 (9)	0.0056 (8)
C3	0.0303 (12)	0.0187 (10)	0.0165 (10)	−0.0002 (9)	0.0053 (9)	0.0077 (8)
C4	0.0348 (13)	0.0183 (10)	0.0120 (10)	0.0007 (9)	0.0030 (9)	0.0045 (8)
C5	0.0298 (12)	0.0143 (9)	0.0134 (10)	0.0015 (8)	0.0031 (8)	0.0046 (8)
C6	0.0228 (11)	0.0149 (9)	0.0109 (9)	0.0000 (8)	−0.0001 (8)	0.0047 (8)
C7	0.0210 (11)	0.0158 (9)	0.0130 (10)	−0.0003 (8)	−0.0018 (8)	0.0041 (8)
C9	0.0245 (11)	0.0192 (10)	0.0140 (10)	0.0025 (8)	0.0026 (8)	0.0053 (8)
C10	0.0214 (11)	0.0204 (11)	0.0216 (11)	0.0037 (8)	0.0022 (9)	0.0053 (9)
C11	0.0247 (11)	0.0209 (11)	0.0205 (11)	0.0030 (9)	−0.0015 (9)	0.0082 (9)
C12	0.0235 (11)	0.0189 (10)	0.0144 (10)	0.0000 (8)	−0.0018 (8)	0.0070 (8)
C13	0.0250 (12)	0.0262 (11)	0.0206 (11)	0.0000 (9)	−0.0027 (9)	0.0087 (9)
C1A	0.0250 (11)	0.0197 (10)	0.0115 (9)	0.0010 (8)	0.0000 (8)	0.0066 (8)
C2A	0.0288 (12)	0.0206 (10)	0.0127 (9)	−0.0014 (9)	−0.0036 (8)	0.0072 (8)
C3A	0.0242 (11)	0.0255 (11)	0.0196 (11)	0.0019 (9)	−0.0014 (9)	0.0086 (9)
C4A	0.0252 (11)	0.0207 (10)	0.0195 (11)	0.0047 (8)	0.0017 (9)	0.0049 (8)
C5A	0.0261 (12)	0.0167 (10)	0.0139 (10)	0.0025 (8)	−0.0009 (8)	0.0054 (8)
C6A	0.0219 (11)	0.0169 (9)	0.0107 (9)	0.0026 (8)	0.0012 (8)	0.0055 (7)
C7A	0.0197 (10)	0.0164 (9)	0.0120 (9)	0.0043 (8)	0.0034 (8)	0.0053 (7)
C9A	0.0271 (11)	0.0175 (10)	0.0130 (10)	−0.0014 (8)	−0.0031 (8)	0.0048 (8)
C10A	0.0244 (11)	0.0225 (11)	0.0171 (10)	−0.0009 (9)	−0.0021 (9)	0.0055 (8)
C11A	0.0251 (12)	0.0201 (10)	0.0184 (11)	−0.0018 (8)	0.0009 (9)	0.0070 (8)
C12A	0.0247 (11)	0.0208 (10)	0.0149 (10)	−0.0005 (9)	0.0023 (8)	0.0072 (8)
C13A	0.0303 (12)	0.0239 (11)	0.0131 (10)	0.0017 (9)	0.0002 (9)	0.0030 (8)
C1B	0.0265 (11)	0.0197 (10)	0.0085 (9)	0.0005 (8)	0.0013 (8)	0.0061 (8)
C2B	0.0257 (11)	0.0211 (10)	0.0138 (10)	0.0017 (9)	0.0036 (8)	0.0055 (8)
C3B	0.0211 (11)	0.0239 (11)	0.0192 (11)	0.0004 (9)	0.0029 (8)	0.0083 (9)
C4B	0.0241 (11)	0.0201 (10)	0.0174 (10)	−0.0017 (8)	−0.0007 (9)	0.0057 (8)
C5B	0.0267 (11)	0.0162 (10)	0.0117 (9)	−0.0013 (8)	0.0006 (8)	0.0049 (8)
C6B	0.0208 (10)	0.0152 (9)	0.0109 (9)	−0.0002 (8)	−0.0014 (8)	0.0049 (7)
C7B	0.0192 (10)	0.0174 (9)	0.0099 (9)	−0.0023 (8)	−0.0027 (7)	0.0054 (7)
C9B	0.0255 (11)	0.0180 (10)	0.0129 (10)	0.0020 (8)	0.0025 (8)	0.0053 (8)
C10B	0.0253 (12)	0.0194 (10)	0.0178 (10)	0.0025 (9)	0.0015 (9)	0.0046 (8)
C11B	0.0218 (11)	0.0188 (10)	0.0181 (10)	0.0015 (8)	−0.0021 (8)	0.0068 (8)
C12B	0.0219 (11)	0.0212 (11)	0.0138 (10)	0.0011 (8)	−0.0025 (8)	0.0074 (8)
C13B	0.0300 (12)	0.0239 (11)	0.0130 (10)	−0.0011 (9)	0.0000 (9)	0.0023 (8)
C1C	0.0225 (11)	0.0165 (10)	0.0128 (10)	0.0018 (8)	0.0006 (8)	0.0050 (8)
C2C	0.0279 (12)	0.0175 (10)	0.0142 (10)	0.0034 (8)	−0.0019 (8)	0.0053 (8)
C3C	0.0325 (12)	0.0188 (10)	0.0129 (10)	0.0033 (9)	−0.0045 (8)	0.0054 (8)
C4C	0.0340 (12)	0.0170 (10)	0.0113 (9)	0.0018 (9)	−0.0027 (9)	0.0040 (8)
C5C	0.0310 (12)	0.0142 (9)	0.0125 (10)	0.0008 (8)	−0.0031 (8)	0.0033 (7)
C6C	0.0220 (10)	0.0148 (9)	0.0101 (9)	−0.0002 (8)	0.0004 (8)	0.0056 (8)
C7C	0.0207 (11)	0.0188 (10)	0.0133 (10)	0.0005 (8)	0.0006 (8)	0.0062 (8)
C9C	0.0267 (12)	0.0193 (10)	0.0133 (10)	−0.0021 (8)	−0.0034 (8)	0.0056 (8)
C10C	0.0261 (12)	0.0204 (11)	0.0213 (11)	−0.0027 (9)	−0.0020 (9)	0.0069 (9)
C11C	0.0263 (12)	0.0214 (11)	0.0218 (11)	−0.0029 (9)	0.0004 (9)	0.0091 (9)
C12C	0.0234 (11)	0.0199 (10)	0.0154 (10)	0.0018 (8)	0.0037 (8)	0.0086 (8)
C13C	0.0254 (12)	0.0278 (12)	0.0191 (11)	0.0016 (9)	0.0039 (9)	0.0086 (9)
N8	0.0268 (10)	0.0172 (8)	0.0138 (8)	0.0030 (7)	0.0031 (7)	0.0073 (7)
N8A	0.0293 (10)	0.0160 (8)	0.0124 (8)	−0.0018 (7)	−0.0021 (7)	0.0072 (7)
N8B	0.0279 (10)	0.0161 (8)	0.0122 (8)	0.0025 (7)	0.0024 (7)	0.0075 (7)
N8C	0.0277 (10)	0.0167 (8)	0.0126 (8)	−0.0017 (7)	−0.0013 (7)	0.0078 (7)
O1	0.0352 (9)	0.0169 (7)	0.0174 (8)	0.0043 (7)	0.0055 (7)	0.0079 (6)
O1A	0.0277 (8)	0.0152 (7)	0.0147 (7)	−0.0004 (6)	−0.0012 (6)	0.0061 (6)
O1B	0.0263 (8)	0.0156 (7)	0.0140 (7)	0.0012 (6)	0.0014 (6)	0.0057 (6)
O1C	0.0348 (9)	0.0169 (7)	0.0165 (8)	−0.0032 (6)	−0.0051 (7)	0.0074 (6)

### Geometric parameters (Å, °)

**Table:**

C1—C13	1.537 (3)	C1B—C6B	1.565 (3)
C1—C2	1.539 (3)	C1B—H1B	0.9800
C1—C6	1.556 (3)	C2B—C3B	1.521 (3)
C1—H1	0.9800	C2B—H2B1	0.9700
C2—C3	1.529 (3)	C2B—H2B2	0.9700
C2—H2A	0.9700	C3B—C4B	1.527 (3)
C2—H2B	0.9700	C3B—H3B1	0.9700
C3—C4	1.525 (3)	C3B—H3B2	0.9700
C3—H3A	0.9700	C4B—C5B	1.529 (3)
C3—H3B	0.9700	C4B—H4B1	0.9700
C4—C5	1.532 (3)	C4B—H4B2	0.9700
C4—H4A	0.9700	C5B—C6B	1.550 (3)
C4—H4B	0.9700	C5B—H5B1	0.9700
C5—C6	1.551 (3)	C5B—H5B2	0.9700
C5—H5A	0.9700	C6B—C7B	1.542 (3)
C5—H5B	0.9700	C6B—C12B	1.548 (3)
C6—C7	1.545 (3)	C7B—O1B	1.239 (3)
C6—C12	1.556 (3)	C7B—N8B	1.350 (3)
C7—O1	1.240 (3)	C9B—N8B	1.460 (3)
C7—N8	1.347 (3)	C9B—C10B	1.515 (3)
C9—N8	1.459 (3)	C9B—H9B1	0.9700
C9—C10	1.518 (3)	C9B—H9B2	0.9700
C9—H9A	0.9700	C10B—C11B	1.519 (3)
C9—H9B	0.9700	C10B—H10E	0.9700
C10—C11	1.525 (3)	C10B—H10F	0.9700
C10—H10A	0.9700	C11B—C12B	1.530 (3)
C10—H10B	0.9700	C11B—H11E	0.9700
C11—C12	1.527 (3)	C11B—H11F	0.9700
C11—H11A	0.9700	C12B—H12E	0.9700
C11—H11B	0.9700	C12B—H12F	0.9700
C12—H12A	0.9700	C13B—H13G	0.9600
C12—H12B	0.9700	C13B—H13H	0.9600
C13—H13A	0.9600	C13B—H13I	0.9600
C13—H13B	0.9600	C1C—C13C	1.534 (3)
C13—H13C	0.9600	C1C—C2C	1.539 (3)
C1A—C2A	1.527 (3)	C1C—C6C	1.560 (3)
C1A—C13A	1.540 (3)	C1C—H1C	0.9800
C1A—C6A	1.566 (3)	C2C—C3C	1.526 (3)
C1A—H1A	0.9800	C2C—H2C1	0.9700
C2A—C3A	1.524 (3)	C2C—H2C2	0.9700
C2A—H2A1	0.9700	C3C—C4C	1.526 (3)
C2A—H2A2	0.9700	C3C—H3C1	0.9700
C3A—C4A	1.523 (3)	C3C—H3C2	0.9700
C3A—H3A1	0.9700	C4C—C5C	1.532 (3)
C3A—H3A2	0.9700	C4C—H4C1	0.9700
C4A—C5A	1.531 (3)	C4C—H4C2	0.9700
C4A—H4A1	0.9700	C5C—C6C	1.549 (3)
C4A—H4A2	0.9700	C5C—H5C1	0.9700
C5A—C6A	1.552 (3)	C5C—H5C2	0.9700
C5A—H5A1	0.9700	C6C—C7C	1.545 (3)
C5A—H5A2	0.9700	C6C—C12C	1.555 (3)
C6A—C7A	1.540 (3)	C7C—O1C	1.241 (3)
C6A—C12A	1.554 (3)	C7C—N8C	1.347 (3)
C7A—O1A	1.241 (3)	C9C—N8C	1.459 (3)
C7A—N8A	1.350 (3)	C9C—C10C	1.520 (3)
C9A—N8A	1.454 (3)	C9C—H9C1	0.9700
C9A—C10A	1.523 (3)	C9C—H9C2	0.9700
C9A—H9A1	0.9700	C10C—C11C	1.527 (3)
C9A—H9A2	0.9700	C10C—H10G	0.9700
C10A—C11A	1.518 (3)	C10C—H10H	0.9700
C10A—H10C	0.9700	C11C—C12C	1.524 (3)
C10A—H10D	0.9700	C11C—H11G	0.9700
C11A—C12A	1.533 (3)	C11C—H11H	0.9700
C11A—H11C	0.9700	C12C—H12G	0.9700
C11A—H11D	0.9700	C12C—H12H	0.9700
C12A—H12C	0.9700	C13C—H13J	0.9600
C12A—H12D	0.9700	C13C—H13K	0.9600
C13A—H13D	0.9600	C13C—H13L	0.9600
C13A—H13E	0.9600	N8—H8	0.8600
C13A—H13F	0.9600	N8A—H8A	0.8600
C1B—C2B	1.531 (3)	N8B—H8B	0.8600
C1B—C13B	1.537 (3)	N8C—H8C	0.8600
			
C13—C1—C2	110.99 (19)	C3B—C2B—C1B	112.89 (18)
C13—C1—C6	112.59 (17)	C3B—C2B—H2B1	109.0
C2—C1—C6	110.66 (17)	C1B—C2B—H2B1	109.0
C13—C1—H1	107.4	C3B—C2B—H2B2	109.0
C2—C1—H1	107.4	C1B—C2B—H2B2	109.0
C6—C1—H1	107.4	H2B1—C2B—H2B2	107.8
C3—C2—C1	112.60 (17)	C2B—C3B—C4B	110.34 (18)
C3—C2—H2A	109.1	C2B—C3B—H3B1	109.6
C1—C2—H2A	109.1	C4B—C3B—H3B1	109.6
C3—C2—H2B	109.1	C2B—C3B—H3B2	109.6
C1—C2—H2B	109.1	C4B—C3B—H3B2	109.6
H2A—C2—H2B	107.8	H3B1—C3B—H3B2	108.1
C4—C3—C2	110.84 (19)	C3B—C4B—C5B	111.08 (18)
C4—C3—H3A	109.5	C3B—C4B—H4B1	109.4
C2—C3—H3A	109.5	C5B—C4B—H4B1	109.4
C4—C3—H3B	109.5	C3B—C4B—H4B2	109.4
C2—C3—H3B	109.5	C5B—C4B—H4B2	109.4
H3A—C3—H3B	108.1	H4B1—C4B—H4B2	108.0
C3—C4—C5	111.46 (18)	C4B—C5B—C6B	113.30 (18)
C3—C4—H4A	109.3	C4B—C5B—H5B1	108.9
C5—C4—H4A	109.3	C6B—C5B—H5B1	108.9
C3—C4—H4B	109.3	C4B—C5B—H5B2	108.9
C5—C4—H4B	109.3	C6B—C5B—H5B2	108.9
H4A—C4—H4B	108.0	H5B1—C5B—H5B2	107.7
C4—C5—C6	113.92 (17)	C7B—C6B—C12B	111.70 (17)
C4—C5—H5A	108.8	C7B—C6B—C5B	108.14 (16)
C6—C5—H5A	108.8	C12B—C6B—C5B	110.97 (17)
C4—C5—H5B	108.8	C7B—C6B—C1B	112.38 (17)
C6—C5—H5B	108.8	C12B—C6B—C1B	106.61 (17)
H5A—C5—H5B	107.7	C5B—C6B—C1B	106.96 (17)
C7—C6—C5	106.96 (17)	O1B—C7B—N8B	118.46 (18)
C7—C6—C1	110.23 (16)	O1B—C7B—C6B	121.51 (18)
C5—C6—C1	109.32 (17)	N8B—C7B—C6B	120.03 (18)
C7—C6—C12	109.54 (17)	N8B—C9B—C10B	114.80 (18)
C5—C6—C12	107.66 (17)	N8B—C9B—H9B1	108.6
C1—C6—C12	112.92 (16)	C10B—C9B—H9B1	108.6
O1—C7—N8	119.01 (19)	N8B—C9B—H9B2	108.6
O1—C7—C6	120.49 (19)	C10B—C9B—H9B2	108.6
N8—C7—C6	120.49 (18)	H9B1—C9B—H9B2	107.5
N8—C9—C10	114.21 (19)	C9B—C10B—C11B	112.40 (19)
N8—C9—H9A	108.7	C9B—C10B—H10E	109.1
C10—C9—H9A	108.7	C11B—C10B—H10E	109.1
N8—C9—H9B	108.7	C9B—C10B—H10F	109.1
C10—C9—H9B	108.7	C11B—C10B—H10F	109.1
H9A—C9—H9B	107.6	H10E—C10B—H10F	107.9
C9—C10—C11	113.44 (19)	C10B—C11B—C12B	113.93 (18)
C9—C10—H10A	108.9	C10B—C11B—H11E	108.8
C11—C10—H10A	108.9	C12B—C11B—H11E	108.8
C9—C10—H10B	108.9	C10B—C11B—H11F	108.8
C11—C10—H10B	108.9	C12B—C11B—H11F	108.8
H10A—C10—H10B	107.7	H11E—C11B—H11F	107.7
C10—C11—C12	115.62 (18)	C11B—C12B—C6B	120.43 (19)
C10—C11—H11A	108.4	C11B—C12B—H12E	107.2
C12—C11—H11A	108.4	C6B—C12B—H12E	107.2
C10—C11—H11B	108.4	C11B—C12B—H12F	107.2
C12—C11—H11B	108.4	C6B—C12B—H12F	107.2
H11A—C11—H11B	107.4	H12E—C12B—H12F	106.9
C11—C12—C6	119.42 (18)	C1B—C13B—H13G	109.5
C11—C12—H12A	107.5	C1B—C13B—H13H	109.5
C6—C12—H12A	107.5	H13G—C13B—H13H	109.5
C11—C12—H12B	107.5	C1B—C13B—H13I	109.5
C6—C12—H12B	107.5	H13G—C13B—H13I	109.5
H12A—C12—H12B	107.0	H13H—C13B—H13I	109.5
C1—C13—H13A	109.5	C13C—C1C—C2C	110.98 (19)
C1—C13—H13B	109.5	C13C—C1C—C6C	112.58 (17)
H13A—C13—H13B	109.5	C2C—C1C—C6C	110.45 (17)
C1—C13—H13C	109.5	C13C—C1C—H1C	107.5
H13A—C13—H13C	109.5	C2C—C1C—H1C	107.5
H13B—C13—H13C	109.5	C6C—C1C—H1C	107.5
C2A—C1A—C13A	109.58 (18)	C3C—C2C—C1C	112.85 (18)
C2A—C1A—C6A	114.28 (17)	C3C—C2C—H2C1	109.0
C13A—C1A—C6A	115.17 (18)	C1C—C2C—H2C1	109.0
C2A—C1A—H1A	105.6	C3C—C2C—H2C2	109.0
C13A—C1A—H1A	105.6	C1C—C2C—H2C2	109.0
C6A—C1A—H1A	105.6	H2C1—C2C—H2C2	107.8
C3A—C2A—C1A	112.93 (19)	C4C—C3C—C2C	110.91 (19)
C3A—C2A—H2A1	109.0	C4C—C3C—H3C1	109.5
C1A—C2A—H2A1	109.0	C2C—C3C—H3C1	109.5
C3A—C2A—H2A2	109.0	C4C—C3C—H3C2	109.5
C1A—C2A—H2A2	109.0	C2C—C3C—H3C2	109.5
H2A1—C2A—H2A2	107.8	H3C1—C3C—H3C2	108.0
C4A—C3A—C2A	110.37 (19)	C3C—C4C—C5C	111.50 (18)
C4A—C3A—H3A1	109.6	C3C—C4C—H4C1	109.3
C2A—C3A—H3A1	109.6	C5C—C4C—H4C1	109.3
C4A—C3A—H3A2	109.6	C3C—C4C—H4C2	109.3
C2A—C3A—H3A2	109.6	C5C—C4C—H4C2	109.3
H3A1—C3A—H3A2	108.1	H4C1—C4C—H4C2	108.0
C3A—C4A—C5A	111.31 (19)	C4C—C5C—C6C	113.84 (17)
C3A—C4A—H4A1	109.4	C4C—C5C—H5C1	108.8
C5A—C4A—H4A1	109.4	C6C—C5C—H5C1	108.8
C3A—C4A—H4A2	109.4	C4C—C5C—H5C2	108.8
C5A—C4A—H4A2	109.4	C6C—C5C—H5C2	108.8
H4A1—C4A—H4A2	108.0	H5C1—C5C—H5C2	107.7
C4A—C5A—C6A	113.14 (18)	C7C—C6C—C5C	106.96 (17)
C4A—C5A—H5A1	109.0	C7C—C6C—C12C	109.73 (18)
C6A—C5A—H5A1	109.0	C5C—C6C—C12C	107.82 (17)
C4A—C5A—H5A2	109.0	C7C—C6C—C1C	109.88 (16)
C6A—C5A—H5A2	109.0	C5C—C6C—C1C	109.36 (18)
H5A1—C5A—H5A2	107.8	C12C—C6C—C1C	112.90 (16)
C7A—C6A—C5A	108.18 (16)	O1C—C7C—N8C	118.87 (19)
C7A—C6A—C12A	111.81 (18)	O1C—C7C—C6C	120.59 (19)
C5A—C6A—C12A	110.80 (17)	N8C—C7C—C6C	120.53 (18)
C7A—C6A—C1A	112.39 (17)	N8C—C9C—C10C	113.87 (18)
C5A—C6A—C1A	106.92 (17)	N8C—C9C—H9C1	108.8
C12A—C6A—C1A	106.65 (17)	C10C—C9C—H9C1	108.8
O1A—C7A—N8A	118.28 (19)	N8C—C9C—H9C2	108.8
O1A—C7A—C6A	121.6 (2)	C10C—C9C—H9C2	108.8
N8A—C7A—C6A	120.09 (19)	H9C1—C9C—H9C2	107.7
N8A—C9A—C10A	114.84 (18)	C9C—C10C—C11C	113.3 (2)
N8A—C9A—H9A1	108.6	C9C—C10C—H10G	108.9
C10A—C9A—H9A1	108.6	C11C—C10C—H10G	108.9
N8A—C9A—H9A2	108.6	C9C—C10C—H10H	108.9
C10A—C9A—H9A2	108.6	C11C—C10C—H10H	108.9
H9A1—C9A—H9A2	107.5	H10G—C10C—H10H	107.7
C11A—C10A—C9A	112.36 (19)	C12C—C11C—C10C	115.43 (19)
C11A—C10A—H10C	109.1	C12C—C11C—H11G	108.4
C9A—C10A—H10C	109.1	C10C—C11C—H11G	108.4
C11A—C10A—H10D	109.1	C12C—C11C—H11H	108.4
C9A—C10A—H10D	109.1	C10C—C11C—H11H	108.4
H10C—C10A—H10D	107.9	H11G—C11C—H11H	107.5
C10A—C11A—C12A	114.16 (19)	C11C—C12C—C6C	119.95 (18)
C10A—C11A—H11C	108.7	C11C—C12C—H12G	107.3
C12A—C11A—H11C	108.7	C6C—C12C—H12G	107.3
C10A—C11A—H11D	108.7	C11C—C12C—H12H	107.3
C12A—C11A—H11D	108.7	C6C—C12C—H12H	107.3
H11C—C11A—H11D	107.6	H12G—C12C—H12H	106.9
C11A—C12A—C6A	120.26 (18)	C1C—C13C—H13J	109.5
C11A—C12A—H12C	107.3	C1C—C13C—H13K	109.5
C6A—C12A—H12C	107.3	H13J—C13C—H13K	109.5
C11A—C12A—H12D	107.3	C1C—C13C—H13L	109.5
C6A—C12A—H12D	107.3	H13J—C13C—H13L	109.5
H12C—C12A—H12D	106.9	H13K—C13C—H13L	109.5
C1A—C13A—H13D	109.5	C7—N8—C9	130.71 (18)
C1A—C13A—H13E	109.5	C7—N8—H8	114.6
H13D—C13A—H13E	109.5	C9—N8—H8	114.6
C1A—C13A—H13F	109.5	C7A—N8A—C9A	132.58 (18)
H13D—C13A—H13F	109.5	C7A—N8A—H8A	113.7
H13E—C13A—H13F	109.5	C9A—N8A—H8A	113.7
C2B—C1B—C13B	109.51 (18)	C7B—N8B—C9B	132.66 (18)
C2B—C1B—C6B	114.23 (17)	C7B—N8B—H8B	113.7
C13B—C1B—C6B	115.45 (18)	C9B—N8B—H8B	113.7
C2B—C1B—H1B	105.6	C7C—N8C—C9C	130.77 (18)
C13B—C1B—H1B	105.6	C7C—N8C—H8C	114.6
C6B—C1B—H1B	105.6	C9C—N8C—H8C	114.6

### Hydrogen-bond geometry (Å, °)

**Table:**

*D*—H···*A*	*D*—H	H···*A*	*D*···*A*	*D*—H···*A*
N8—H8···O1B	0.86	2.11	2.967 (2)	173
N8B—H8B···O1	0.86	2.03	2.868 (2)	166
N8A—H8A···O1C	0.86	2.03	2.872 (2)	165
N8C—H8C···O1A	0.86	2.10	2.959 (2)	172
